# Feasibility and Preliminary Efficacy of Digital Interventions for Depressive Symptoms in Working Adults: Multiarm Randomized Controlled Trial

**DOI:** 10.2196/41590

**Published:** 2023-06-16

**Authors:** Rachael Wallis Taylor, Rhian Male, Marcos Economides, Heather Bolton, Kate Cavanagh

**Affiliations:** 1 Unmind Ltd London United Kingdom; 2 University of Sussex Sussex United Kingdom

**Keywords:** depression, digital intervention, randomized controlled trial, RCT, cognitive behavioral therapy, CBT, acceptance and commitment therapy, ACT, behavioral activation, BA, mobile phone

## Abstract

**Background:**

Depressive symptoms are highly prevalent and have broad-ranging negative implications. Digital interventions are increasingly available in the workplace context, but supporting evidence is limited.

**Objective:**

This study aimed to evaluate the feasibility, acceptability, and preliminary efficacy of 3 digital interventions for depressive symptoms in a sample of UK-based working adults experiencing mild to moderate symptoms.

**Methods:**

This was a parallel, multiarm, pilot randomized controlled trial. Participants were allocated to 1 of 3 digital interventions or a waitlist control group and had 3 weeks to complete 6 to 8 short self-guided sessions. The 3 interventions are available on the Unmind mental health app for working adults and draw on behavioral activation, cognitive behavioral therapy, and acceptance and commitment therapy. Web-based assessments were conducted at baseline, postintervention (week 3), and at 1-month follow-up (week 7). Participants were recruited via Prolific, a web-based recruitment platform, and the study was conducted entirely on the web. Feasibility and acceptability were assessed using objective engagement data and self-reported feedback. Efficacy outcomes were assessed using validated self-report measures of mental health and functioning and linear mixed models with intention-to-treat principles.

**Results:**

In total, 2003 individuals were screened for participation, of which 20.22% (405/2003) were randomized. A total of 92% (373/405) of the participants were retained in the study, 97.4% (295/303) initiated their allocated intervention, and 66.3% (201/303) completed all sessions. Moreover, 80.6% (229/284) of the participants rated the quality of their allocated intervention as excellent or good, and 79.6% (226/284) of the participants were satisfied or very satisfied with their intervention. All active groups showed improvements in well-being, functioning, and depressive and anxiety symptoms compared with the control group, which were maintained at 4 weeks. Hedges *g* effect sizes for depressive symptoms ranged from −0.53 (95% CI −0.25 to −0.81) to −0.74 (95% CI −0.45 to −1.03).

**Conclusions:**

All interventions were feasible and acceptable, and the preliminary efficacy findings indicated that their use may improve depressive symptoms, well-being, and functioning. The predefined criteria for a definitive trial were met.

**Trial Registration:**

International Standard Randomised Controlled Trial Number (ISRCTN) ISRCTN13067492; https://www.isrctn.com/ISRCTN13067492

## Introduction

Depression is primarily characterized by low mood, reduced functioning, and poor quality of life [1,2]. Although major depression has been the focus of much previous research, mild to moderate depressive symptoms are highly prevalent and are estimated to occur in approximately 20% to 22% of the general population across Europe, Asia, and the United States [3-5]. In addition, mild to moderate depression has been established as a precursor to and risk factor for major depression [6]. Here, we use the terms “mild” and “moderate” in line with the categories described by Kroenke et al [7] (and defined as those scoring between 5 and 14 on the Patient Health Questionnaire-8 [PHQ-8]). Mild to moderate depression can result in reduced functioning and both presenteeism and absenteeism in the workplace [8]. The economic costs associated with mild to moderate depression are thought to approach those of major depression [9], and it is now widely recognized that employers have a responsibility to support the mental health of their employees [1,10,11]. However, traditional face-to-face or therapist-guided psychological interventions, although effective [12,13], are not easily scalable in the workplace context and are subject to stigma and a perception of inefficacy [14,15]. Therefore, there is a considerable need for accessible, scalable interventions with established efficacy to be made available to working adults experiencing mild to moderate depressive symptoms.

Digital interventions can be scaled at low cost [16], and smartphone apps that deliver content designed to alleviate the symptoms of common mental health problems are now widely available. However, evidence regarding their impact is still emerging [17], and only a small proportion of commercially developed mental health apps have established efficacy [18,19], suggesting the need for more methodologically robust trials. A recent meta-analysis evaluating digital health interventions for depression included 83 randomized controlled trials (RCTs) and showed a significant medium overall effect versus control [20], whereas another meta-analysis focusing only on mobile interventions for depressive symptoms and including just 10 studies also reported moderate symptom reduction versus control [21]. The included programs were predominantly based on cognitive behavioral therapy (CBT) techniques [22], whereas other evidence-based therapeutic models for depression (such as acceptance and commitment therapy [ACT] and behavioral activation [BA]) were underrepresented and require more thorough evaluation. For example, a recent meta-analysis of digital ACT interventions for depression found limited evidence for reliable clinical changes in symptoms, and most interventions were therapist guided [23], suggesting a need for further evaluation of self-guided digital ACT programs. In addition, a recent overview of systematic reviews found that most digital interventions for depression are at least 6 weeks long [17], with less evidence for shorter, more condensed interventions, such as those included in this study. Finally, digital mental health interventions specifically designed for delivery in the workplace have not been widely evaluated, although evidence indicates that they can be effective in improving psychological well-being and work effectiveness [24].

This need for a broader evaluation of digital interventions for depression is further demonstrated by the reportedly low user adherence to such programs [25] and mixed findings for the acceptability of web-based mental health interventions in the workplace [26]. Given the association between adherence to digital interventions for depression and clinical outcomes [27], it is important to establish feasibility and acceptability. This is in line with recommendations from the UK Medical Research Council and the National Institute for Health Research on evaluating complex interventions, which state that interventions can be undermined by poor acceptability, compliance, and delivery and definitive trials evaluating efficacy can be subject to poor recruitment and retention [28,29]. An initial pilot study can establish feasibility and acceptability and allow for necessary adjustments to both intervention and study design before carrying out a definitive trial to determine efficacy or evaluating the specific dissemination of these interventions in real-world workplace settings.

Therefore, we conducted a pilot RCT evaluating the feasibility, acceptability, and preliminary efficacy of 3 brief, self-guided, stand-alone digital interventions for low mood and depressive symptoms available on the Unmind mental health platform. Unmind is a web-based and mobile app for working adults that features a range of content designed to help employees measure, manage, and improve their mental health and well-being. Although employees are granted access to Unmind via their employer, it can be used both within and outside the workplace. Unmind has previously been shown to be feasible and acceptable when used by healthy participants to manage symptoms of stress and anxiety or boost resilience [30], but it has not been evaluated for use by individuals with mild to moderate depression. As depression has been shown to negatively impact intervention engagement [31], we deemed it necessary to establish acceptability and report on app engagement in this novel study population. In addition, because the Unmind interventions in this study differ substantially from those previously evaluated, we deemed it important to evaluate them independently and not assume equivalency. Finally, as difficulties in recruiting participants into depression trials are very common [32], we aimed to test whether a previously used recruitment method would be feasible for this novel study population.

The Unmind interventions evaluated in this study are underpinned by BA [33], CBT [22], or ACT [34], three evidence-informed psychological therapies for depression. The Unmind app deliberately includes interventions that use different therapeutic modalities, giving the user free choice over which modality feels the most relevant and appealing to them. This is important, as a recent meta-analysis suggests that giving users autonomy and choice over intervention parameters leads to greater adherence and better clinical outcomes [35]. Thus, this study aimed to evaluate the feasibility and acceptability of the 3 interventions independently (using an efficient multiarm design) without directly comparing them.

Participants were randomized to 1 of the 3 interventions or to a waitlist control group. A waitlist design was selected to ensure that all participants were provided access to the evidence-based interventions evaluated. This approach was deemed the most appropriate for establishing intervention feasibility and acceptability, which was the primary aim of this study, as it does not require the use of an active control comparator. The study was not powered to evaluate efficacy outcomes; therefore, a passive control design with the potential to capture both specific and nonspecific intervention effects was considered the most appropriate for establishing preliminary indications of efficacy. Each intervention was assessed according to the predefined progression criteria for a definitive trial in a community-based sample of working adults experiencing mild to moderate depressive symptoms.

## Methods

### Trial Preregistration

This trial was conducted in line with the CONSORT (Consolidated Standards of Reporting Trials) 2010 guidelines, including extensions for pilot studies and multiarm trials [36-38], and reported in accordance with the CONSORT-EHEALTH (CONSORT of Electronic and Mobile Health Applications and Online Telehealth) checklist [39]. The study protocol was preregistered on ISRCTN (International Standard Randomised Controlled Trial Number; registration number ISRCTN13067492) and the Open Science Framework [40].

### Ethics Approval, Informed Consent, and Participation

The trial was conducted according to the guidelines of the Declaration of Helsinki, and all procedures involving human participants were approved by the Sciences & Technology Cluster Research Ethics Committee at the University of Sussex (ethics reference number ER/KC226/4). Written informed consent was obtained from all trial participants. Participants received GB £10 (US $10.16) for completing each study assessment and GB £1 (US $1.16) for completing a brief screening assessment. All study data were anonymous, and a full description of the data protection procedures is included in the preregistered trial protocol.

### Design and Setting

This study was a parallel, multiarm, pilot RCT with preassessment (time point 0 [baseline]; *t*0), postassessment (time point 1 [week 3]; *t*1), and follow-up assessment (time point 2 [week 6]; *t*2). Participants were randomly allocated to 1 of the 3 brief self-guided psychological interventions for low mood and depressive symptoms on the Unmind platform or to a waitlist control group in a 1:1:1:1 allocation ratio without stratification. All study procedures were conducted on the web in the United Kingdom.

### Participants and Procedure

Eligible participants (1) were aged ≥18 years; (2) were based in the United Kingdom; (3) were fluent in English; (4) were currently in full-time or part-time employment; (5) had access to the internet; (6) had an active Prolific account; and (7) scored between 5 and 14 on the PHQ-8 (refer to the *Secondary Outcome Measures* section [41]), indicating mild to moderate depressive symptoms. Additional criteria included interest in, and willingness to use, the study interventions and willingness to be randomized. Individuals reporting a diagnosis of bipolar disorder, schizophrenia or other psychotic spectrum disorder, alcohol or substance use disorder, or neurocognitive disorder were excluded. Current engagement with psychological therapy for low mood or depression via a health care professional and current or previous engagement with the Unmind platform or an Unmind study were not permitted.

Participants were recruited via Prolific, and all study assessments were completed on the web via Qualtrics. Prolific is a web-based recruitment platform that has been empirically tested across key attributes such as response rates and data quality [42]. It allows researchers to advertise studies to a large and diverse participant pool (>119,000 as of February 2023), with existing demographic information per individual that can be used for initial screening. Prolific is available for individuals aged at least 18 years from most Organization for Economic Co-operation and Development countries, and participants undergo various verification and technical (eg, IP address) checks before acceptance on the platform. Individuals who met Prolific’s automated initial screening criteria (based in the United Kingdom, fluent in English, employed, and having not participated in previous studies assessing the Unmind platform) were invited to take part in a study to evaluate the impact of completing 1 of the several interventions (each of which is comprised of between 6 and 8 sessions) for symptoms of low mood, featured on the Unmind mental health app. Following informed consent, participants were directed to a brief screening questionnaire to assess study-specific eligibility criteria. Study screening was separated by sex and age bracket, with the aim of recruiting a sample demographically similar to the UK working population. Eligibility was assessed using a script programmed by the authors that automatically checked all participant responses against the eligibility criteria.

All eligible individuals were then invited to complete the baseline assessment (*t*0), which was preceded by the full study consent form and included a battery of measures to assess current mental health and well-being as well as demographic and treatment history data. The baseline assessment was closed once the desired sample size was achieved. Participants were then randomized into 1 of the 4 groups via the Qualtrics “randomizer” feature, which uses block randomization with variable block sizes to generate balanced groups. It was not possible to blind the participants to group assignments. The research team remained blind to the group assignment for the duration of data collection but was unblinded during the analysis. At the end of the baseline assessment, participants assigned to the intervention arms were presented with an instruction video explaining how to access their intervention, including the use of a unique anonymous ID to sign up on the Unmind platform. A written version of these instructions was also provided via Prolific. Participants then had 3 weeks to complete their allocated intervention and were sent a weekly intervention reminder message via Prolific’s anonymous messaging system. At the end of the intervention period, access to the Unmind platform was withdrawn. Participants were then sent an email invitation to complete the post (*t*1) assessment and a further email invitation 4 weeks after for the final follow-up (*t*2) assessment, via Prolific. Each invitation was followed by a reminder message 3 days later. The *t*1 and *t*2 assessments included measures of mental health and well-being conducted at *t*0 plus a feedback questionnaire that included questions adapted from the Mobile App Rating Scale (MARS) [43] (intervention groups only, *t*1). On the completion of *t*2, the control group was given access to the Unmind platform for 3 weeks.

### Interventions

#### Overview

**Figure 1:**
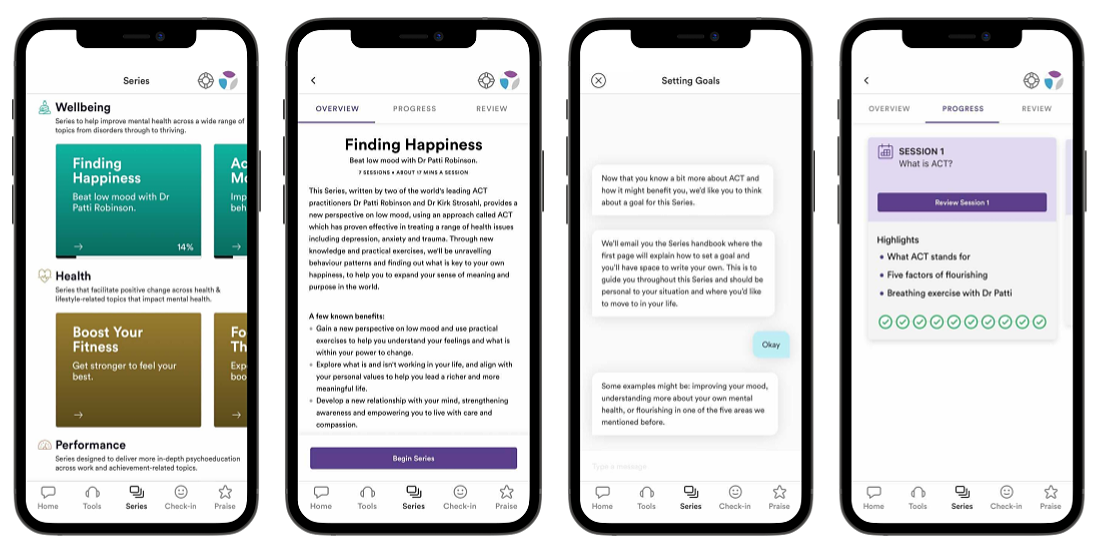
Screenshots of the Finding Happiness course on the Unmind platform.

Unmind is a digital platform designed to help working adults measure, manage, and improve their mental health and well-being and has previously been described in detail [30]. This study evaluated 3 brief interventions (known as courses) available on the Unmind platform, accessible via web-based or mobile apps, intended to help users manage and improve low mood and depressive symptoms and designed to be completed over the course of several days or weeks (Figure 1). The versions of the Unmind app that were live during the 3-week intervention period were 2.90.0 to 2.92.0, and no major app changes or updates were launched. Participants had access to a modified version of the Unmind platform with only their allocated intervention available:

#### Activate Your Mood

Activate Your Mood (AYM) is a BA-based course consisting of eight 10-minute sessions designed to help the user understand the links between their behavior and mood and to increase their levels of activity, with the aim of improving mood. Activities encouraged between sessions include a mood diary, activity monitoring, and activity scheduling. Participants were advised to complete 1 session every other day.

#### Mind Your Mood

Mind Your Mood (MYM) is rooted in CBT for depression and consists of six 10-minute sessions. The course encourages the user to understand the link between cognition and mood and empowers them to spot and challenge negative thoughts. Advised activities between sessions include spotting and challenging negative thoughts and tackling rumination.

#### Finding Happiness

Finding Happiness (FH) is an ACT-based course and consists of 7 sessions of 10 to 18 minutes each. By examining behavior, clarifying values, and designing experiments, FH aims to help users expand their sense of meaning and purpose in the world, thereby improving their mood. Each session includes an experiential exercise (eg, mindfulness), and users were encouraged to practice these between sessions.

### Outcomes

#### Primary Outcome Measures

In line with the guidelines for pilot studies evaluating complex interventions [44,45], the following primary outcomes were assessed via a combination of objective data (including app use data) and self-report feedback data collected at *t*1 and *t*2. Feedback data were largely based on questions adapted from the MARS—the most widely used, validated scale for evaluating the quality and content of mental health apps [43].

- Feasibility: recruitment rate, intervention uptake, and retention (at *t*1 and *t*2)
- Acceptability: intervention adherence (completion rate, defined as the proportion of participants completing all sessions of their allocated course within the intervention period), activity adherence (the self-reported completion rate for all additional intervention activities to be completed between sessions), participant satisfaction (“How satisfied are you with the Unmind Course you were asked to complete overall?”), and reasons for nonadherence (2 multiselect questions)
- Engagement: average intervention sessions completed in each group, average number of between-session activities completed, and select questions adapted from sections A and B of the MARS at *t*1
- Transferability of the intervention to other settings or populations: assessed by select questions adapted from section E of the MARS at *t*1
- Relevance: assessed using subjective feedback data gathered at *t*1 (“Would you agree that the Unmind Course was relevant to your personal experience?”)
- Bad effects: the proportion of participants who reported experiencing bad effects or lasting bad effects from the intervention (as described in the study by Carlisle et al [35]) and the proportion of participants whose PHQ-8 scores increased above the minimally clinically important difference for the PHQ-8 (≥5 points [46]), assessed at *t*1 and *t*2

#### Secondary Outcome Measures

To determine the preliminary efficacy of each intervention, changes in depression and anxiety symptoms, well-being, and health-related productivity loss were assessed as secondary outcomes using data collected at *t*0, *t*1 (primary end point), and *t*2 via the following validated questionnaires:

- The PHQ-8 [41] is an 8-item scale that screens for the presence and severity of depression, with scores ranging from 0 to 24. The PHQ-8 has excellent internal consistency (Cronbach α=.89) and excellent test-retest reliability [47].
- The Generalized Anxiety Disorder-7 (GAD-7) scale [48] is a 7-item scale that screens for the presence and severity of an anxiety disorder, with scores ranging from 0 to 21. The GAD-7 has excellent reliability and internal consistency (Cronbach α=.89 [48,49]).
- The Short Warwick-Edinburgh Mental Well-being Scale (SWEMWBS) [50], a short 7-item version of the original 14-item Warwick-Edinburgh Mental Well-being Scale, was designed to measure aspects of mental well-being primarily related to functioning [51]. The scores range from 7 to 35. The SWEMWBS has high internal consistency (Cronbach α=.84 in the general population).
- The Unmind Index is a 26-item self-report measure designed to capture the key components of positive mental well-being and specific symptoms of mental illness [52]. Scores were normalized and standardized to population norms, with a mean of 100 and an SD of 15. The Unmind Index shows excellent internal consistency for overall well-being (McDonald hierarchical ω=0.92) and all subscales (Cronbach α=.83-.92).
- The Work Productivity and Activity Impairment Scale (WPAI) questionnaire [53] is a 6-item self-report measure of health-related work productivity loss for the employed population. This study used the specific health problem version (WPAI:SHP), which asks respondents questions concerning impairment owing to “low mood.” The WPAI provides 4 scores, indicating the degree of absenteeism, presenteeism (impairment while working), overall work impairment (the impact of both absenteeism and presenteeism), and overall activity impairment owing to low mood. Scores are expressed as percentages, and the WPAI has demonstrated sufficient construct validity and test-retest reliability (with correlation coefficients ranging from 0.71-0.87 for overall productivity loss) [53].

### Progression Criteria

Progression criteria for a definitive trial were predefined in line with recent recommendations [54] and included full recruitment within 1 month, ≥30% intervention completion rates for interventions, ≥50% of participants reporting being “satisfied” or “very satisfied” with the brief intervention and rating the quality as “good” or “excellent,” and between-group effect sizes for secondary outcomes including at least a small effect (Hedges *g* ≥0.2) for change in PHQ-8 scores at *t*1 (intervention vs control).

### Statistical Analyses

A sample size calculation indicated that approximately 100 participants per group were required to estimate feasibility outcomes with a margin of error ≤10%, based on a conservative estimate of 50% for retention and adherence. This is consistent with guidelines suggesting that 60 to 100 participants per intervention arm are optimal for estimating binary outcomes in pilot studies, such as the recruitment and completion rate outlined in the *Progression Criteria* section [55]. We, therefore, aimed to recruit 400 participants. The PHQ-8 score distribution of a previous UK study sample recruited via Prolific was used as a reference to estimate the appropriate number of participants required for screening (n=2000; [52]).

The primary outcomes were reported using descriptive statistics and appropriate measures of central tendency, and Fisher exact or ANOVA tests were used to compare between groups, as appropriate. Objective in-app use data were provided by Unmind, the creator of the interventions evaluated. For simplicity, the intervention sessions were only characterized as complete if all components of the session were played. Thus, for each participant, the intervention engagement ranged from 0 sessions complete to all sessions complete. Use data also included the duration of each session completed by each participant. Descriptive statistics were used to characterize engagement and stratify participants according to whether they completed, started but did not complete, or failed to start their allocated intervention.

The secondary efficacy outcomes were assessed using an intention-to-treat (ITT) approach (in which all randomized participants were included in the statistical analysis, regardless of intervention engagement or attrition) and linear mixed effects (LME) models. Each LME included “time point” as a within-subjects factor (levels: *t*0, *t*1, and *t*2), “group” as a between-subjects factor, and their interaction as fixed effects, with a separate baseline for each participant. The time point was coded as a categorical variable to avoid enforcing a linear relationship with outcomes. For each model fit, residuals were checked via quantile-quantile plots to assess the model assumptions and goodness of fit. For each outcome, we plotted the estimated marginal means (EMMs) for each time point and intervention arm with SEs and reported between-group contrasts (with 95% CIs) comparing changes from *t*0 to *t*1 and *t*0 to *t*2 for each intervention arm relative to the control group (with *P* values). We also reported a standardized effect size (Hedges *g* with 95% CI) for each between-group contrast. Hedges *g* was calculated using EMMs and pooled SDs. The 95% CIs were calculated using equations 15 and 16 from the study by Nakagawa and Cuthill [56].

Exploratory analyses examined the proportion of individuals that experienced a clinically important change in PHQ-8 score, defined according to Löwe et al [57] as a change of ≥5 from baseline and a change in subscale scores for the Unmind Index, as per the study protocol.

Owing to a technical error, planned per-protocol analyses of the secondary outcomes could not be conducted. All other preregistered primary and secondary outcomes were reported, except for the analysis of qualitative feedback. The protocol deviations are detailed in Multimedia Appendix 1.

## Results

### Participants

**Figure 2:**
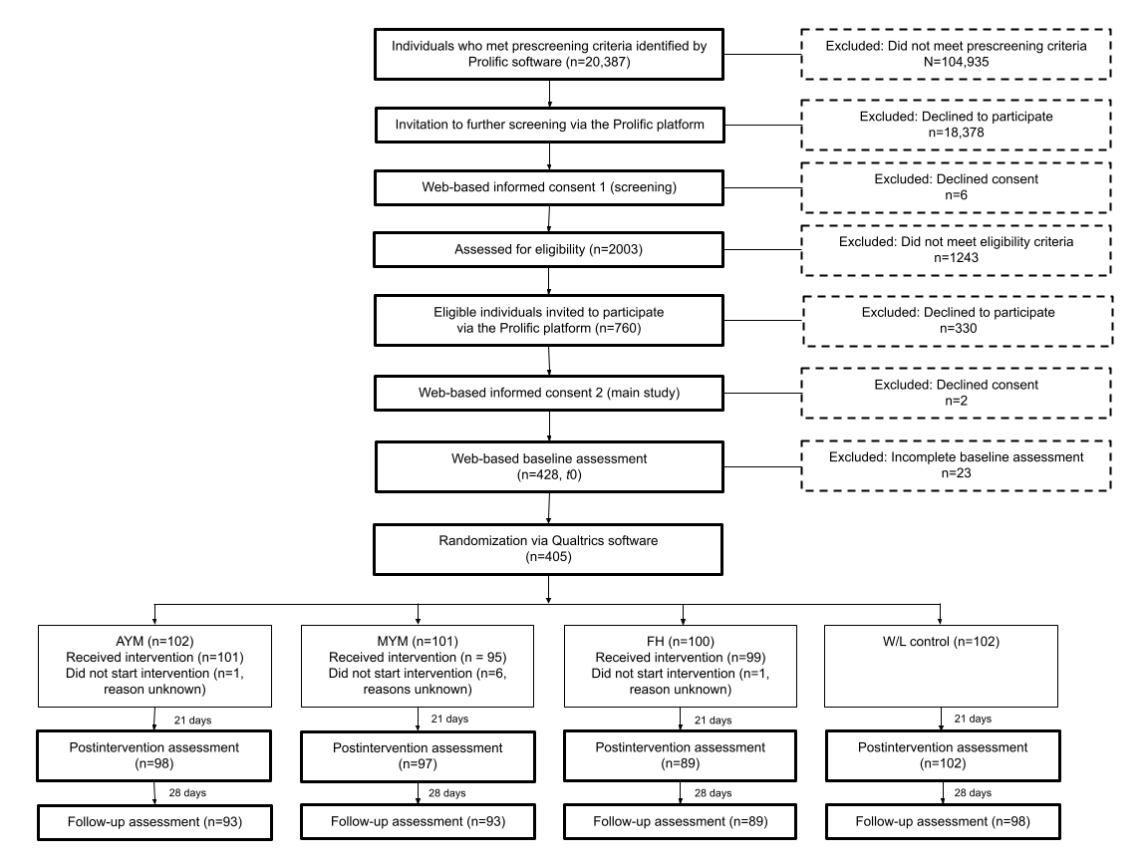
CONSORT (Consolidated Standards of Reporting Trials) flow diagram. AYM: Activate Your Mood; FH: Finding Happiness; MYM: Mind Your Mood; W/L: waitlist; t0: time point 0 (baseline).

Recruitment and data collection took place between September and November 2021. A total of 405 eligible participants completed the baseline survey (Figure 2). The demographics and use of other interventions are presented in Table 1. The sample was 54.8% (222/405) female, 88.1% (357/405) White, and had a mean age of 36.9 (SD 9.5) years, which is reasonably consistent with the UK general population [58]. Baseline scores on measures of mental health, well-being, and functioning are presented in Table 2 and indicate that, on average, participants experienced mild symptoms of depression and anxiety and had below-average well-being when compared with population norms [41,48], and the majority of the participants had not accessed treatment in the last 6 months. WPAI scores showed impaired work and activity functioning owing to low mood but low rates of absenteeism. The baseline scores on the subscales of the Unmind Index are shown in Multimedia Appendix 2.

**Table 1:** Participant demographics and intervention use.

Variable			Overall (n=405)		Study arm						
					Activate Your Mood (n=102)		Mind Your Mood (n=101)		Finding Happiness (n=100)		Control (n=102)
Age (years), mean (SD; range)			36.9 (9.5; 18.0-67.0)		35.7 (8.8; 18.0-62.0)		36.7 (10.7; 20.0-67.0)		38.2 (9.6; 19.0-63.0)		36.9 (8.7; 19.0-59.0)
**Sex, n (%)**											
	Female	222 (54.8)		61 (59.8)		51 (50.5)		54 (54)		56 (54.9)	
	Male	183 (45.2)		41 (40.2)		50 (49.5)		46 (46)		46 (45.1)	
**Gender identity same as registered sex at birth, n (%)**											
	Yes	404 (99.8)		101 (99)		101 (100)		100 (100)		102 (100)	
**Ethnicity, n (%)**											
	Asian	22 (5.4)		6 (5.9)		8 (7.9)		3 (3)		5 (4.9)	
	Black	13 (3.2)		6 (5.9)		1 (1)		3 (3)		3 (2.9)	
	Mixed or multiple	13 (3.2)		6 (5.9)		4 (4)		0 (0)		3 (2.9)	
	White	357 (88.1)		84 (82.4)		88 (87.1)		94 (94)		91 (89.2)	
**Employment, n (%)**											
	Full-time employee	282 (69.6)		70 (68.6)		68 (67.3)		71 (71)		73 (71.6)	
	Part-time employee	83 (20.5)		21 (20.6)		22 (21.8)		19 (19.0)		21 (20.6)	
	Self-employed or contractor	40 (9.9)		11 (10.8)		11 (10.9)		10 (10)		8 (7.8)	
**Industry, n (%)**											
	Agriculture, forestry, or mining	4 (1)		1 (1)		1 (1)		0 (0)		2 (2)	
	Industrials	25 (6.2)		6 (5.9)		7 (6.9)		8 (8)		4 (3.9)	
	Energy or utilities	8 (2)		0 (0)		2 (2)		2 (2)		4 (3.9)	
	Transport or logistics	13 (3.2)		3 (2.9)		3 (3)		3 (3)		4 (3.9)	
	Media or creative industries	15 (3.7)		5 (4.9)		5 (5)		5 (5)		0 (0)	
	Data infrastructure or telecommunications	9 (2.2)		2 (2)		3 (3)		1 (1)		3 (2.9)	
	Health care	58 (14.3)		19 (18.6)		14 (13.9)		15 (15)		10 (9.8)	
	Education	74 (18.3)		18 (17.6)		13 (12.9)		15 (15)		28 (27.5)	
	Life sciences	8 (2)		3 (2.9)		2 (2)		2 (2)		1 (1)	
	Retail or e-commerce	35 (8.6)		6 (5.9)		9 (8.9)		14 (14)		6 (5.9)	
	Hospitality, food, leisure, or travel	15 (3.7)		6 (5.9)		3 (3)		3 (3)		3 (2.9)	
	Public or social service	67 (16.5)		13 (12.7)		20 (19.8)		19 (19)		15 (14.7)	
	Finance, insurance, or real estate	39 (9.6)		9 (8.8)		11 (10.9)		9 (9)		10 (9.8)	
	Professional services	35 (8.6)		11 (10.8)		8 (7.9)		4 (4)		12 (11.8)	
**Long-term leave, n (%)**											
	Yes (general sick leave)	3 (0.7)		1 (1)		0 (0)		1 (1)		1 (1)	
	Yes (sick leave due to low mood)	2 (0.5)		0 (0)		N/A^a^		1 (1)		1 (1)	
	Yes (any other reason)	8 (2)		0 (0)		3 (3)		4 (4)		1 (1)	
**Education, n (%)**											
	No formal qualifications	1 (0.2)		0 (0)		0 (0)		1 (1)		0 (0)	
	High school education	148 (36.5)		37 (36.3)		35 (34.7)		42 (42)		34 (33.3)	
	University degree	162 (40)		47 (46.1)		40 (39.6)		39 (39)		36 (35.3)	
	Postgraduate degree	94 (23.2)		18 (17.6)		26 (25.7)		18 (18)		32 (31.4)	
**Intervention use in the last 6 months, n (%)**											
	Use of mental health apps	36 (8.9)		11 (10.8)		8 (7.9)		5 (5)		12 (11.8)	
	Accessed employer support	13 (3.2)		2 (2)		3 (3)		7 (7)		1 (1)	
	Accessed health care professional support	34 (8.4)		12.0 (11.8)		9 (8.9)		7 (7)		6 (5.9)	
	Use of antidepressants	51 (12.6)		16.0 (15.7)		16 (15.8)		8 (8)		11 (10.8)	
	Accessed psychological therapy	36 (8.9)		13 (12.7)		9 (8.9)		7 (7)		7 (6.9)	
	Accessed other interventions	21 (5.2)		7 (6.9)		4 (4)		7 (7)		3 (2.9)	
**Intervention use at baseline, n (%)**											
	Use of mental health apps	9 (2.2)		2 (2)		2 (2)		2 (2)		3 (2.9)	
	Use of antidepressants	47 (11.6)		14 (13.7)		14 (13.9)		8 (8)		11 (10.8)	
	Accessing other interventions	8 (2)		3 (2.9)		2 (2)		1 (1)		2 (2)	
	Accessing other self-help resources	25 (6.2)		7 (6.9)		8 (8)		5 (5)		5 (5)	

^a^N/A: not applicable.

**Table 2:** Baseline mental health, well-being, and functioning scores.

Variable			Overall (n=405)		Study arm						
					Activate Your Mood (n=102)		Mind Your Mood (n=101)		Finding Happiness (n=100)		Control (n=102)
**Patient Health Questionnaire-8**											
	Value, mean (SD)	8.7 (2.6)		8.6 (2.7)		8.7 (2.4)		9.1 (2.6)		8.6 (2.7)	
	Value, median (IQR; range)	8.0 (7.0-11.0; 5.0-14.0)		8.0 (6.0-11.0; 5.0-14.0)		8.0 (7.0-11.0; 5.0-14.0)		9.0 (7.0-11.0; 5.0-14.0)		8.0 (6.0-11.0; 5.0-14.0)	
**Generalized Anxiety Disorder-7**											
	Value, mean (SD)	7.6 (4.0)		7.7 (3.8)		7.7 (3.7)		7.3 (4.0)		7.8 (4.5)	
	Value, median (IQR; range)	7.0 (5.0-10.0; 0.0-20.0)		7.0 (5.0-10.0; 1.0-19.0)		7.0 (5.0-10.0; 2.0-18.0)		7.0 (4.8-9.0; 0.0-20.0)		7.0 (4.0-10.8; 1.0-20.0)	
**Short Warwick-Edinburgh Mental Well-being Scale**											
	Value, mean (SD)	19.4 (2.5)		19.3 (2.4)		19.4 (2.5)		19.3 (2.3)		19.8 (2.8)	
	Value, median (IQR; range)	19.3 (18.0-20.7; 13.3-29.3)		18.9 (18.0-20.0; 13.3-27.0)		19.3 (17.4-20.7; 14.1-25.0)		19.3 (17.4-20.7; 15.3-26.0)		19.3 (18.0-21.5; 14.8-29.3)	
**Unmind Index**											
	Value, mean (SD)	93.8 (7.4)		93.2 (6.8)		93.6 (7.6)		93.3 (6.9)		95.2 (8.0)	
	Value, median (IQR; range)	93.4 (89.0-98.1; 74.5-117.2)		93.4 (87.9-97.3; 77.8-111.7)		92.6 (89.5-98.5; 75.4-113.7)		92.8 (89.0-98.0; 74.5-114.6)		95.8 (90.1-99.1; 79.4-117.2)	
**WPAI^a^ absenteeism**											
	Value, mean (SD)	4.2 (11.5)		3.9 (11.8)		5.3 (13.9)		4.0 (9.5)		3.6 (10.4)	
	Value, median (IQR; range)	0.0 (0.0-2.6; 0.0-100.0)		0.0 (0.0-2.7; 0.0-100.0)		0.0 (0.0-1.4; 0.0-100.0)		0.0 (0.0-3.6; 0.0-54.5)		0.0 (0.0-1.7; 0.0-75.0)	
**WPAI presenteeism**											
	Value, mean (SD)	41.2 (20.3)		43.5 (21.6)		39.5 (20.4)		39.9 (18.2)		41.8 (20.8)	
	Value, median (IQR; range)	40.0 (30.0-60.0; 0.0-90.0)		40.0 (30.0-60.0; 0.0-80.0)		40.0 (30.0-55.0; 0.0-90.0)		40.0 (30.0-50.0; 0.0-90.0)		40.0 (30.0-60.0; 0.0-90.0)	
**WPAI work impairment**											
	Value, mean (SD)	42.6 (21.3)		44.8 (22.2)		41.1 (21.7)		41.4 (19.7)		42.9 (21.5)	
	Value, median (IQR; range)	40.0 (30.0-60.0; 0.0-97.5)		46.2 (30.0-61.9; 0.0-87.5)		40.0 (30.0-57.5; 0.0-92.0)		40.0 (30.0-53.7; 0.0-92.7)		40.0 (30.0-60.0; 0.0-97.5)	
**WPAI activity impairment**											
	Value, mean (SD)	44.1 (21.7)		45.2 (20.5)		45.8 (22.6)		43.4 (20.9)		42.1 (22.8)	
	Value, median (IQR; range)	40.0 (30.0-60.0; 0.0-100.0)		50.0 (30.0-60.0; 0.0-80.0)		50.0 (30.0-60.0; 10.0-100.0)		40.0 (30.0-60.0; 0.0-90.0)		40.0 (20.0-60.0; 0.0-90.0)	

^a^WPAI: Work Productivity and Activity Impairment Scale.

### Primary Outcomes

#### Feasibility (Recruitment and Retention)

The participants were recruited in September 2021. A total of 760 eligible individuals were identified, and 405 individuals completed the baseline assessment within 48 hours. The intervention uptake and retention across the study and the reasons for ineligibility are shown in Figure 2. Overall, 95.3% (386/405) of the participants completed the *t*1 assessment, and 92.1% (373/405) of the participants completed the *t*2 assessment. The intervention update was high, with only 8 participants failing to initiate their assigned intervention across the 3 groups. Retention was significantly different between the study groups at *t*1 (*P*=.002), with FH being the lowest (89/100, 89%). There was no difference in retention between the groups at *t*2 (*P*=.27).

#### Acceptability and Engagement (Adherence, Satisfaction, and Feedback)

The objective adherence data obtained from the Unmind platform database are summarized in Table 3. Overall, 97.4% (295/303) of the participants started their allocated intervention, and 66.3% (201/303) of the participants completed all relevant sessions. Of those who initiated their intervention, 68.1% (201/295) went on to complete it. The mean time spent on sessions was 72.3 (SD 34.6) minutes (Table 3). This differed significantly between the intervention groups (*F*_2,292_=77.95; *P*<.001). Intervention completers spent a mean of 86.9 (SD 25.3) minutes engaging with sessions, again with a significant difference between the groups (*F*_2,192_=3019; *P*<.001). The mean number of days taken to complete was 14.3 (SD 5.7), with a significant difference between the groups (*F*_2,192_=8.21; *P*<.001; Table 3). Completion of the between-session activities was self-reported (Table 3). Overall, 70.4% (200/284) of the participants reported completing all recommended activities between sessions, with no significant difference between the groups (*P*=.30).

The subjective feedback data for the intervention group participants completing the *t*1 assessment are presented in Table 4. Overall, 79.6% (226/284) of the participants reported being either “satisfied” or “very satisfied” with their allocated course, 56.3% (160/284) of the participants rated the course design as “moderately” or “highly interesting and fun,” and 61.9% (176/284) of the participants rated the course content as “moderately” or “highly interesting and fun.” In total, 90.8% (258/284) of the participants said that they were able to use the app immediately or that it was easy to learn how to use it. In total, 80.6% (229/284) of the participants agreed that the quality of their allocated course was either “good” or “excellent.” Ratings did not significantly differ between the groups (all *P*>.05; Table 4). The reported reasons for session and activity noncompletion are shown in Multimedia Appendix 3.

**Table 3:** Intervention adherence.

Variable		Overall (n=303)	Study arm		
			AYM^a^ (n=102)	MYM^b^ (n=101)	FH^c^ (n=100)
**Started intervention^d^**					
	Participants, n	295	101	95	99
	Participants randomized, % (95% CI)	97.4 (94.9-98.9)	99 (94.7-100.0)	94.1 (87.5-97.8)	99 (94.6-100.0)
**Completers^d^**					
	Participants, n	201	59	74	68
	Participants randomized, % (95% CI)	66.3 (60.7-71.6)	57.8 (47.7-67.6)	73.3 (63.5-81.6)	68 (57.9-77.0)
	Participants starting intervention, % (95% CI)	68.1 (62.6-73.2)	58.4 (48.2-68.1)	77.9 (68.2-85.8)	68.7 (58.6-77.6)
	Days to complete intervention, mean (SD)	14.3 (5.7)	16.7 (4.3)	13.4 (4.9)	13.0 (6.9)
	Time (minutes) on sessions, mean (SD)	86.9 (25.3)	76.3 (1.6)	64.2 (0.8)	120.1 (7.4)
**Partial completers^d^**					
	Participants, n	94	42	21	31
	Participants randomized, % (95% CI)	31.9 (26.8-37.4)	41.2 (31.5-51.4)	20.8 (13.4-30.0)	31 (22.1-41.0)
Number of sessions completed (all participants)^d^, mean (SD)		5.6 (2.3)	6.0 (2.7)	5.2 (1.7)	5.7 (2.4)
Time on course sessions (all participants)^d^, mean (SD)		72.3 (34.6)	59.5 (23.3)	56.0 (17.2)	100.9 (38.8)
**Completed all activities**					
	Participants, n	200	74	68	58
	Participants randomized, % (95% CI)	66 (60.4-71.3)	72.5 (62.8-80.9)	66.7 (62.8-80.9)	58 (47.7-67.8)
	Participants starting intervention, % (95% CI)	67.8 (62.1-73.1)	73.3 (63.5-81.6)	71.6 (61.4-80.4)	58.6 (48.2-68.4)

^a^AYM: Activate Your Mood.

^b^MYM: Mind Your Mood.

^c^FH: Finding Happiness.

^d^Objective adherence data obtained from the Unmind platform database.

**Table 4:** Participant feedback.

Participant feedback		Overall (n=284; time point 1), n (%)	AYM^a^ (n=98), n (%)	MYM^b^ (n=97), n (%)	FH^c^ (n=89), n (%)	*P* value^d^	
**Design of intervention**							.66
	Dull, not fun, or interesting at all	2 (0.7)	0 (0)	1 (1.0)	1 (1.1)		
	Mostly boring	19 (6.7)	5 (5.1)	7 (7.2)	7 (7.9)		
	Okay, fun enough	103 (36.3)	37 (37.8)	32 (33.0)	34 (38.2)		
	Moderately interesting and fun	123 (43.3)	42 (42.9)	48 (49.5)	33 (37.1)		
	Highly interesting and fun	37 (13.0)	14 (14.3)	9 (9.3)	14 (15.7)		
**Content of intervention**							.32
	Dull, not fun, or interesting at all	3 (1.1)	0 (0)	1 (1.0)	2 (2.2)		
	Mostly boring	20 (7.0)	3 (3.1)	7 (7.2)	10 (11.2)		
	Okay, fun enough	85 (29.9)	30 (30.6)	32 (33.0)	23 (25.8)		
	Moderately interesting and fun	124 (43.7)	47 (48.0)	37 (38.1)	40 (44.9)		
	Highly interesting and fun	52 (18.3)	18 (18.4)	20 (20.6)	14 (15.7)		
**Ease of intervention use**							.22
	No (limited instructions, confusing menu labels or icons, and complicated)	5 (1.8)	2 (2.0)	3 (3.1)	0 (0)		
	Usable after a lot of time and effort	5 (1.8)	1 (1.0)	3 (3.1)	1 (1.1)		
	Usable after some time and effort	16 (5.6)	9 (9.2)	3 (3.1)	4 (4.5)		
	Easy to learn how to use the app	124 (43.7)	38 (38.8)	39 (40.2)	47 (52.8)		
	Able to use the app immediately	134 (47.2)	48 (49.0)	49 (50.5)	37 (41.6)		
**Likelihood of recommending intervention**							.93
	I would not recommend it to anyone	9 (3.2)	3 (3.1)	4 (4.1)	2 (2.2)		
	There are very few people I would recommend it to	23 (8.1)	8 (8.2)	7 (7.2)	8 (9.0)		
	There are several people whom I would recommend it to	105 (37.0)	35 (35.7)	39 (40.2)	31 (34.8)		
	There are many people I would recommend it to	77 (27.1)	29 (29.6)	21 (21.6)	27 (30.3)		
	I would recommend it to everyone experiencing low mood	70 (24.6)	23 (23.5)	26 (26.8)	21 (23.6)		
**Relevance of intervention**							.61
	Strongly agree	54 (19.0)	15 (15.3)	21 (21.6)	18 (20.2)		
	Agree	162 (57.0)	61 (62.2)	54 (55.7)	47 (52.8)		
	Neither agree nor disagree	41 (14.4)	12 (12.2)	15 (15.5)	14 (15.7)		
	Disagree	19 (6.7)	6 (6.1)	4 (4.1)	9 (10.1)		
	Strongly disagree	8 (2.8)	4 (4.1)	3 (3.1)	1 (1.1)		
**Satisfaction with intervention**							.34
	Very satisfied	83 (29.2)	33 (33.7)	22 (22.7)	28 (31.5)		
	Satisfied	143 (50.4)	47 (48.0)	57 (58.8)	39 (43.8)		
	Neither satisfied nor dissatisfied	47 (16.5)	15 (15.3)	15 (15.5)	17 (19.1)		
	Dissatisfied	10 (3.5)	3 (3.1)	2 (2.1)	5 (5.6)		
	Very dissatisfied	1 (0.4)	0 (0)	1 (1.0)	0 (0)		
**Quality of intervention**							.85
	Excellent	109 (38.4)	39 (39.8)	34 (35.1)	36 (40.4)		
	Good	120 (42.3)	40 (40.8)	44 (45.4)	36 (40.4)		
	Okay	52 (18.3)	19 (19.4)	18 (18.6)	15 (16.9)		
	Poor	3 (1.1)	0 (0)	1 (1.0)	2 (2.2)		
Bad effects in intervention (yes)		3 (1.1)	0 (0)	2 (2.1)	1 (1.1)	.42	
Bad effects at follow-up (yes)		2 (0.7)	1 (1.1)	1 (1.1)	0 (0)	.41	

^a^AYM: Activate Your Mood.

^b^MYM: Mind Your Mood.

^c^FH: Finding Happiness.

^d^Fisher exact test.

#### Transferability and Relevance

The perceived transferability and relevance of the interventions were assessed using the *t*1 subjective feedback questionnaire. Overall, 51.8% (147/284) of the participants completing the assessment said that they would recommend their allocated intervention to many or all people if they were experiencing low mood, and 76.1% (216/284) of the participants agreed or strongly agreed that it was relevant to them. The ratings are summarized in Table 4 and did not differ significantly by group.

#### Bad Effects

In total, 98.9% (281/284) of the intervention group participants completing the postintervention assessment reported no bad effects at *t*1, and 99.3% (282/284) of those completing the *t*2 assessment reported no lasting bad effects, with no significant difference between the groups (*t*1: *P*=.42 and *t*2: *P*=.41; Table 4). Increases in PHQ-8 scores meeting the definition of meaningful deterioration (an increase ≥5 from baseline) were also assessed as a measure of bad effects. In total, 1% (1/98), 3% (3/97), and 5% (4/89) of the participants completing the *t*1 assessment met the criteria in each of the intervention groups (AYM, MYM, and FH*,* respectively). Moreover, 5% (5/93), 5% (5/93), and 2% (2/89) of the participants met the criteria at follow-up. In contrast, 12.7% (13/102) of the control group participants completing the *t*1 assessment met the criteria for a clinically significant increase in scores, and 12% (12/98) of the control group participants met the criteria at *t*2. Fisher test showed that the difference between groups was significant at both time points (*P*=.001 and *P*=.04). Post hoc omnibus tests showed the difference to be between the control group and each of the intervention arms at *t*1 (AYM: *P*=.001; MYM: *P*=.004; FH: *P*<.001) and between the control group and FH at *t*2 (*P*=.08).

### Secondary Outcomes

**Figure 3:**
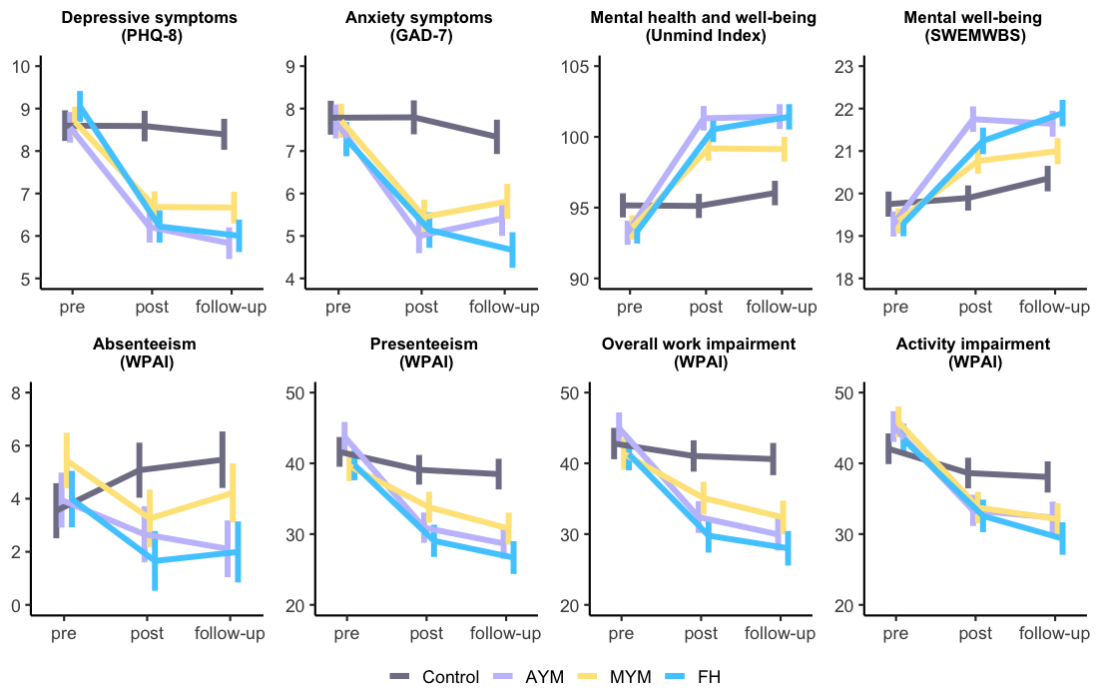
Plots showing estimated marginal means obtained from linear mixed effects models for secondary efficacy outcomes. Error bars represent SE of the mean. AYM: Activate Your Mood; FH: Finding Happiness; GAD-7: Generalized Anxiety Disorder-7; MYM: Mind Your Mood; PHQ-8: Patient Health Questionnaire-8; SWEMWBS: Short Warwick-Edinburgh Mental Well-being Scale; WPAI: Work Productivity and Activity Impairment Scale.

Changes in measures of mood, anxiety, well-being, and functioning were evaluated for the ITT sample. EMMs obtained from the LME analyses for each outcome measure are shown in Figure 3. The contrast and between-group effect sizes are presented in Table 5. All intervention groups reported a significant reduction in depression (PHQ-8) and anxiety (GAD-7) scores at *t*1, maintained at *t*2, and a significant increase in well-being (SWEMWBS and Unmind Index) at *t*1, maintained at *t*2, when compared with the control group (all *P*<.05; Table 5). The between-group Hedges *g* effect sizes were in the medium range for change in depression and anxiety scores for all intervention groups at *t*1 and the small to medium ranges at *t*2. Large effects were identified for change in well-being according to the SWEMWBS or Unmind Index for the AYM and FH intervention groups at *t*1 and maintained at *t*2 according to the Unmind Index (Table 5).

All 3 intervention groups reported a significant reduction in activity impairment owing to low mood at *t*1, which was maintained at *t*2 (all *P*<.05; Table 5). Both the AYM and FH groups reported a significant reduction in presenteeism and overall work impairment at *t*1, which was maintained at *t*2 (all *P*<.05). Some significant reductions in absenteeism were reported (*P*=.03; Table 5), but a large proportion of participants who completed the WPAI reported no absenteeism at baseline for the previous week (277/382, 72.5%). The between-group Hedges *g* effect sizes were predominantly small at *t*1 and *t*2 for WPAI outcomes (Table 5).

**Table 5:** Linear mixed effects model contrasts and between-group (intervention vs control) effect sizes for secondary efficacy outcomes.

Outcome			*t*1^a^ minus *t*0^b^							*t*2^c^ minus *t*0				
			Estimate (SE; 95% CI)		*P* value		Hedges *g* (95% CI)		Estimate (SE; 95% CI)			*P* value		Hedges *g* (95% CI)
**Patient Health Questionnaire-8**														
	AYM^d^	−2.34 (0.53; −3.36 to −1.31)		<.001		−0.62 (−0.91 to −0.34)		−2.52 (0.53; −3.57 to −1.48)			<.001		−0.66 (−0.95 to −0.38)	
	MYM^e^	−1.99 (0.53; −3.02 to −0.96)		<.001		−0.53 (−0.81 to −0.25)		−1.81 (0.53; −2.85 to −0.77)			<.001		−0.48 (−0.76 to −0.20)	
	FH^f^	−2.82 (0.54; −3.86 to −1.77)		<.001		−0.74 (−1.03 to −0.45)		−2.84 (0.54; −3.90 to −1.79)			<.001		−0.74 (−1.03 to −0.46)	
**Generalized Anxiety Disorder-7**														
	AYM	−2.71 (0.52; −3.72 to −1.69)		<.001		−0.73 (−1.01 to −0.44)		−1.83 (0.53; −2.87 to −0.80)			<.001		−0.49 (−0.77 to −0.21)	
	MYM	−2.28 (0.52; −3.29 to −1.26)		<.001		−0.61 (−0.90 to −0.33)		−1.45 (0.53; −2.48 to −0.42)			.006		−0.38 (−0.66 to −0.11)	
	FH	−2.15 (0.53; −3.19 to −1.11)		<.001		−0.57 (−0.85 to −0.29)		−2.16 (0.53; −3.21 to −1.12)			<.001		−0.57 (−0.85 to −0.29)	
**Short Warwick-Edinburgh Mental Well-being Scale**														
	AYM	2.32 (0.41; 1.5 to 3.1)		<.001		0.80 (0.52 to 1.09)		1.76 (0.41; 1.0 to 2.6)			<.001		0.60 (0.32 to 0.88)	
	MYM	1.27 (0.41; 0.47 to 2.1)		.002		0.44 (0.16 to 0.72)		1.04 (0.41; 0.23 to 1.9)			.01		0.35 (0.08 to 0.63)	
	FH	1.80 (0.41; 1.0 to 2.6)		<.001		0.61 (0.33 to 0.90)		2.00 (0.42; 1.2 to 2.8)			<.001		0.68 (0.39 to 0.96)	
**Unmind Index**														
	AYM	8.12 (1.13; 5.92 to 10.33)		<.001		1.01 (0.72 to 1.3)		7.33 (1.15; 5.09 to 9.57)			<.001		0.89 (0.61 to 1.18)	
	MYM	5.62 (1.13; 3.41 to 7.83)		<.001		0.70 (0.41 to 0.98)		4.67 (1.15; 2.42 to 6.91)			<.001		0.57 (0.29 to 0.85)	
	FH	7.25 (1.15; 5.00 to 9.50)		<.001		0.89 (0.59 to 1.18)		7.22 (1.16; 4.96 to 9.49)			<.001		0.88 (0.59 to 1.17)	
**WPAI^g^ absenteeism**														
	AYM	−2.82 (1.68; −6.10 to 0.47)		.09		−0.23 (−0.51 to 0.04)		−3.76 (1.71; −7.09 to −0.43)			.03		−0.31 (−0.59 to −0.03)	
	MYM	−3.71 (1.70; −7.02 to −0.40)		.03		−0.31 (−0.59 to −0.03)		−3.14 (1.73; −6.53 to 0.24)			.07		−0.25 (−0.53 to 0.02)	
	FH	−3.86 (1.73; −7.24 to −0.47)		.03		−0.31 (−0.59 to −0.03)		−3.91 (1.77; −7.36 to −0.47)			.03		−0.31 (−0.59 to −0.03)	
**WPAI presenteeism**														
	AYM	−10.31 (3.34; −16.82 to −3.79)		.002		−0.43 (−0.71 to −0.15)		−11.93 (3.39; −18.56 to −5.31)			<.001		−0.49 (−0.77 to −0.21)	
	MYM	−3.28 (3.36; −9.83 to 3.29)		.33		−0.14 (−0.41 to 0.14)		−5.69 (3.44; −12.40 to 1.03)			.10		−0.23 (−0.51 to 0.05)	
	FH	−8.19 (3.43; −14.89 to −1.48)		.02		−0.34 (−0.62 to −0.06)		−9.94 (3.51; −16.78 to −3.10)			.005		−0.40 (−0.68 to −0.12)	
**WPAI overall impairment**														
	AYM	−10.82 (3.42; −17.49 to −4.16)		.002		−0.44 (−0.72 to −0.16)		−12.84 (3.48; −19.62 to −6.05)			<.001		−0.52 (−0.80 to −0.24)	
	MYM	−4.49 (3.44; −11.20 to 2.22)		.19		−0.18 (−0.46 to 0.09)		−6.80 (3.52; −13.67 to 0.08)			.05		−0.27 (−0.55 to 0.01)	
	FH	−9.75 (3.52; −16.61 to −2.89)		.006		−0.39 (−0.67 to −0.11)		−11.09 (3.59; −18.10 to −4.09)			.002		−0.44 (−0.72 to −0.15)	
**WPAI activity impairment**														
	AYM	−8.42 (3.34; −14.94 to −1.90)		.01		−0.35 (−0.63 to −0.08)		−8.87 (3.39; −15.49 to −2.25)			.009		−0.37 (−0.64 to −0.09)	
	MYM	−8.66 (3.35; −15.20 to −2.12)		.01		−0.36 (−0.64 to −0.08)		−9.74 (3.40; −16.36 to −3.11)			.004		−0.40 (−0.68 to −0.12)	
	FH	−7.40 (3.41; −14.05 to −0.75)		.03		−0.31 (−0.59 to −0.03)		−10.05 (3.43; −16.74 to −3.36)			.003		−0.41 (−0.69 to −0.13)	

^a^t1: time point 1 (week 3).

^b^t0: time point 0 (baseline).

^c^t2: time point 2 (week 6).

^d^AYM: Activate Your Mood.

^e^AYM: Mind Your Mood.

^f^FH: Finding Happiness.

^g^WPAI: Work Productivity and Activity Impairment Scale.

### Subgroup Analyses and Exploratory Outcomes

#### Clinically Important Change

A total of 36.5% (148/405) of participants had a baseline PHQ-8 score >9, indicating that they would likely meet criteria for depression, and were therefore evaluated for clinically important change, defined as a change in score ≥5 points as per the study by Löwe et al [57]. In the AYM group, 41% (14/34) of the participants reached the threshold for clinically significant change at *t*1, increasing to 53% (18/34) by follow-up. In total, 35% (13/37) of the MYM participants reached a clinically significant change at *t*1, which increased to 41% (15/37) at *t*2. Moreover, 44% (17/39) of the FH participants reached this threshold by *t*1, which decreased slightly to 41% (16/39) at *t*2. Only 8% (3/39) of the control group experienced a clinically significant change by *t*1, which increased to 21% (8/39) at *t*2. There was a significant difference in the rates of clinical change between the groups at *t*1 (*P*=.002) and *t*2 (*P*=.04). Post hoc tests showed the difference to be between the control group and each of the intervention arms at *t*1 and the control group and AYM at *t*2.

#### Change in Unmind Index Subscale Scores

The Unmind Index is a measure of mental health and well-being, comprising 7 subscales [52]. Changes in the subscale scores were investigated as exploratory outcomes in the ITT sample using LMEs. Significantly greater changes in subscale scores were identified for each course versus control at the postintervention assessment and were maintained at follow-up (all *P*<.05). The only nonsignificant interaction was on the Sleep subscale (MYM vs control at *t*2; *P*=.07), and effect sizes were predominantly in the small to medium range (Multimedia Appendices 4 and 5).

## Discussion

### Principal Findings

This study evaluated the feasibility, acceptability, and preliminary efficacy of 3 brief digital interventions for depressive symptoms in working adults. Participants were a mildly symptomatic, largely untreated group, with below-average well-being and considerably impaired workplace functioning but low absenteeism when compared with other populations experiencing depressive symptoms [8]. All 3 interventions were found to be feasible and acceptable to this group. This was a pilot study and therefore not powered for formal hypothesis testing, but significant improvements in mental health and well-being were observed for all intervention groups compared with the waitlist control group. The between-group effect sizes ranged from small to large, and all predefined progression criteria for a definitive trial were met.

Engagement with the study interventions was high, with an overall completion rate of 66.3%, and participants spent an average of >70 minutes engaging with their allocated intervention. In addition, 70.4% (200/284) of the participants self-reported completing all additional between-session activities. This completion rate exceeded the minimum of 30% specified in our progression criteria and was higher than the 53% completion rate reported in a recent meta-analysis of digital interventions for depression, where the average number of intervention sessions was 7.3, which is comparable with this study [20]. However, the completion rate differed by intervention group and was the lowest for AYM (58%). Possible reasons for this include differences in intervention design, with AYM consisting of 8 short sessions, compared with MYM and FH, which comprise 6 and 7 sessions, respectively. AYM was the only intervention to advise a 1-day gap between sessions and contained the most extensive program of between-session activities. The reported reasons for session noncompletion support the notion that the additional time commitment associated with AYM contributed to fewer participants completing all relevant sessions. The lack of time, forgetting to engage, and reduced motivation were the most frequently reported reasons for activity noncompletion, which is in line with previous reports of barriers to engagement with digital mental health interventions [59].

Despite the differences in completion rates between interventions, there were no significant differences in subjective feedback ratings. Interestingly, ratings pertaining to how “interesting or fun” participants found the intervention content and design were considerably lower than ratings of intervention quality, ease of use, and overall satisfaction. This discrepancy may, at least in part, be because of the nature of depressive symptoms, which can include reduced capacity for interest and enjoyment and nonreactivity of mood [2]. Therefore, the ratings of interest and fun may have captured participant symptomatology rather than intervention acceptability. Although these interventions were not necessarily intended to be “fun,” increasing participant interest through an improved app design may be one area in which acceptability and engagement could be maximized in the future. Very few bad effects were reported, and the proportion of participants in the intervention groups that experienced a clinically important increase in scores was lower than the expected level of deterioration following in-person psychotherapy [60].

Preliminary efficacy findings showed a significant reduction in depressive symptoms for all 3 intervention groups when compared with a waitlist control group, with improvements maintained at the 4-week follow-up. The between-group Hedges *g* effect sizes were predominantly in the medium range, with CIs ranging from small to large. Although preliminary, this is broadly in line with several recent meta-analyses that evaluated digital interventions for symptoms of depression [17,20,61]. However, there are several reasons for the promising effect sizes reported in this study. First, studies that focus on mild to moderate depression and those that include self-guided interventions (without human support) tend to report smaller effect sizes [20,21]. Second, most trials included in previous meta-analyses involved longer interventions, suggesting that the current interventions may have a similar impact at a smaller effective dose. Finally, the moderate effect size for symptoms of depression following the FH intervention compares favorably with previous evaluations of ACT-based interventions that largely report small effects [23].

In addition to symptoms of depression, all intervention groups reported significant reductions in anxiety compared with the control group, and between-group effect sizes were in the medium range and maintained at follow-up. Mental well-being also significantly improved for all intervention groups, with the Unmind Index showing large effects for FH and AYM. To date, few studies on digital interventions for depressive symptoms have included well-being as an outcome, making it difficult to compare these findings with previous work, although some studies have reported small beneficial effects on quality of life. Capturing well-being outcomes may be important for understanding the broader impact of interventions beyond diagnostic symptomatology, as it provides insight into a wider range of constructs related to mental health than diagnostic tests alone [52], and mental well-being is known to have a considerable impact on various health and social outcomes [62]. Importantly, the replication of these analyses in a definitive trial is necessary to determine whether a sustained improvement in overall well-being can be achieved with digital interventions for depressive symptoms.

Reported rates of presenteeism, overall work impairment, and impairment in regular activity were significantly improved at both postintervention and follow-up for 2 of the 3 interventions. This is broadly consistent with previous studies reporting small improvements in workplace functioning following digital mental health interventions for employees [24,63]. However, few studies have specifically evaluated the impact of digital interventions for depression on workplace outcomes, suggesting a need for further research. Significant effects were also identified for absenteeism, but given the very low baseline rate, the results should be interpreted with caution and require replication. Depression is reported to be the medical condition with the greatest negative impact on time management and productivity [64], and presenteeism accounts for most of the associated cost to employers [65,66]. If replicated in a definitive trial, the reductions in presenteeism identified for FH and AYM could have important economic implications, the extent of which should be examined in the context of a definitive RCT. Although changes in presenteeism for the MYM group did not reach significance in this pilot study, scores did reduce over the study period, and changes in functioning can lag behind clinical outcomes [67]. Therefore, further evaluation of changes in presenteeism and other measures of functioning in a definitive trial is warranted for all 3 intervention groups.

The implementation of interventions specifically targeting individuals with early, milder depressive symptoms—known as indicated prevention—could help prevent symptoms from developing into more severe depressive episodes, offering personal benefit to affected individuals and reducing costs to employers. Although early evidence suggests that indicated prevention interventions for depression can produce small beneficial effects [68], outcomes in workplace settings have been mixed [69]. The findings of this study, although preliminary, suggest that the interventions evaluated in this study could have potential preventative effects by reducing the progression of depressive symptoms among employees with mild to moderate symptoms. However, further research is required to test the efficacy and effectiveness of these interventions in workplace settings and to use more rigorous diagnostic tools to test for the incidence of depression.

The Unmind app includes 3 interventions designed to tackle depressive symptoms based on CBT, BA, or ACT to provide users with choice over which therapeutic modality they feel appeals most to them. Providing a choice of interventions has been found to be associated with improved clinical outcomes and greater engagement [35,70] and thus may further ensure that the needs of adults with depressive symptoms are met. In future versions of the Unmind app, it might be possible to ask users questions that can enable the app to recommend a specific therapeutic modality deemed most suited to them, via built-in algorithms. Although a meta-analysis of psychotherapies for adult depression found generally comparable effects for these 3 modalities, the authors reported high levels of heterogeneity and a high risk of bias for most studies [71]. In addition, in a recent meta-analysis of digital interventions for depression, 74% of the included trials were based on CBT, suggesting that evidence for other therapeutic modalities is still emerging [20]. Indeed, recent meta-analyses of ACT-based [23] and BA-based [61] digital interventions concluded that more evidence is needed. Studies such as this study, which include interventions that use a variety of therapeutic modalities, may therefore help improve our understanding of non–CBT-based digital interventions for depression.

### Strengths and Limitations

This study has several strengths. All procedures were preregistered, and the CONSORT guidelines were followed throughout. Participant retention was very high, meaning there was little missing data and, therefore, a low risk of bias in our estimations of intervention effects. Intervention engagement was measured objectively, which is important given the evidence of inflated rates of self-reported digital intervention use in a study setting [72]. All measures of mental health, well-being, and functioning were validated by self-report assessments, and the intervention feedback questions were adapted from an established assessment of mobile app quality.

However, this study recruited participants exclusively from Prolific; therefore, participants were a self-selected group likely to be highly motivated to engage with research and open to using a digital, app-based intervention. In addition, the completion of the study assessments was incentivized. Therefore, it remains unknown whether the findings of this study can be generalized to the wider UK working population experiencing symptoms of depression. The extent to which the wider population would be open to using app-based interventions remains unknown. In addition, it was not possible to blind participants to group allocation, as is usually the case with web-based trials. To capture feasibility and acceptability outcomes, we adapted questions from the original expert-rater version of the MARS rather than the newer user-specific version [73], which would have been a more appropriate means of gathering intervention feedback in this study. To reduce participant burden, we also included only a subset of questions from the MARS that were deemed to be the most relevant and not the full scale. This included feasibility outcomes identified as being important for evaluating complex interventions [45] but excluded factors such as intervention esthetics.

We also noted that absenteeism rates in our study sample were low at baseline, and regression model diagnosis using quantile-quantile plots indicated that the residuals were not normally distributed. Therefore, any efficacy findings pertaining to absenteeism should be interpreted with caution. We were also unable to conduct planned exploratory analyses to evaluate the impact of intervention engagement because of a technical error. This study used a waitlist, passive control group design, meaning that inferences cannot be made about intervention mechanisms of action at this stage, although we note that such an evaluation was not the aim of this study. Furthermore, this was a pilot trial with the primary aim of evaluating intervention feasibility and acceptability and was not powered for formal hypothesis testing. Therefore, replication of the efficacy analyses reported in this study in a larger definitive RCT is essential, and the efficacy findings in this study should be interpreted with appropriate caution. Finally, although this study recruited working adults, the study interventions were not evaluated in a workplace setting, and it remains unknown whether the findings reported in this study would translate to such settings. Further evaluation using real-world effectiveness trials is required.

### Conclusions

This pilot study demonstrates the feasibility and acceptability of 3 brief digital interventions available on the Unmind app to help tackle the low mood and depressive symptoms experienced by working adults. Preliminary efficacy findings indicate that the use of the interventions evaluated may support improvements in depression, anxiety, well-being, and functioning, according to validated self-report measures. The between-group effect sizes associated with the interventions were broadly in line with those reported in the existing RCT literature examining self-guided digital interventions for depression. Indications of significantly reduced presenteeism provide a rationale for further examination of changes in functioning and the possible associated economic implications for employers. Importantly, a definitive trial to establish the efficacy of these digital interventions is warranted, as all predefined progression criteria were met.

## Appendix

Multimedia Appendix 1 Protocol deviations.

Multimedia Appendix 2 Baseline scores on the subscales of the Unmind Index measure of mental health and well-being.

Multimedia Appendix 3 Reported reasons for session and activity noncompletion, obtained at t1 (time point 1; week 3).

Multimedia Appendix 4 Plots of estimated marginal means obtained from the linear mixed effects models for the 7 subscales of the Unmind Index.

Multimedia Appendix 5 Contrasts and between-group (intervention vs control) effect size calculations from linear mixed effects models applied to each of the 7 Unmind Index subscales.

Multimedia Appendix 6 CONSORT-eHEALTH checklist (V 1.6.1).

## Abbreviations

ACT: acceptance and commitment therapy
AYM: Activate Your Mood
BA: behavioral activation
CBT: cognitive behavioral therapy
CONSORT: Consolidated Standards of Reporting Trials
CONSORT-EHEALTH: Consolidated Standards of Reporting Trials of Electronic and Mobile Health Applications and Online Telehealth
EMM: estimated marginal mean
FH: Finding Happiness
GAD-7: Generalized Anxiety Disorder-7
ISRCTN: International Standard Randomised Controlled Trial Number
ITT: intention-to-treat
LME: linear mixed effects
MARS: Mobile App Rating Scale
MYM: Mind Your Mood
PHQ-8: Patient Health Questionnaire-8
RCT: randomized controlled trial
SWEMWBS: Short Warwick-Edinburgh Mental Well-being Scale
t0: time point 0 (baseline)
t1: time point 1 (week 3)
t2: time point 2 (week 6)
WPAI: Work Productivity and Activity Impairment Scale

## Data Availability

The data sets generated and analyzed during this study are available from the corresponding author on reasonable request.
