# Supplementary material for: Feasibility and Preliminary Efficacy of Digital Interventions for Depressive Symptoms in Working Adults: Multiarm Randomized Controlled Trial
Source: JMIR Form Res. 2023 Jun 16;7:e41590. doi: 10.2196/41590 (PMC10337296; doi:10.2196/41590)
Supplement: Multimedia Appendix 1 [file formative_v7i1e41590_app1.docx]

**Multimedia Appendix 1**

Protocol deviations

Due to a technical error, planned per protocol analyses and a planned exploratory analysis evaluating the relationship between intervention adherence and pre-post change in secondary outcome measures could not be carried out. The error entailed accidental removal of participants’ unique anonymous ID from the Unmind database after study completion, which meant that intervention adherence data (platform data) was no longer linked to participants’ unique anonymous ID. Thus, participants’ intervention adherence data could no longer be matched to their responses to secondary outcome measures. The planned per-protocol analyses would have involved assessing the preliminary efficacy of the three intervention arms, only including participants that completed >75% of sessions in their allocated intervention.

We also did not examine the impact of prior or concurrent use of treatment interventions on secondary outcomes as use was reportedly low in this sample.

We also opted not to report on analysis of qualitative feedback as we deemed this to be beyond the scope of this manuscript.

All other planned analyses were conducted and reported, and no other protocol deviations occurred.
