# Supplementary material for: Feasibility and Preliminary Efficacy of Digital Interventions for Depressive Symptoms in Working Adults: Multiarm Randomized Controlled Trial
Source: JMIR Form Res. 2023 Jun 16;7:e41590. doi: 10.2196/41590 (PMC10337296; doi:10.2196/41590)
Supplement: Multimedia Appendix 2 [file formative_v7i1e41590_app2.docx]

| **Multimedia Appendix 2.** Baseline scores on subscales of the Unmind Index measure of mental health and wellbeing | | | | | |
| --- | --- | --- | --- | --- | --- |
| Variable | | Study arm | | | |
|  | Overall, n = 405 | AYM, n = 102 | MYM, n = 101 | FH, n = 100 | Control, n = 102 |
| **Calmness** |  |  |  |  |  |
| Mean (SD) | 96.2 (12.0) | 95.9 (11.4) | 97.0 (10.9) | 96.1 (12.6) | 95.7 (13.1) |
| Range | 68.7, 124.0 | 68.7, 124.0 | 68.7, 124.0 | 68.7, 121.2 | 68.7, 124.0 |
| Median (IQR) | 96.0 (87.7, 104.3) | 96.0 (87.7, 104.3) | 96.0 (87.7, 104.3) | 96.0 (87.7, 104.3) | 93.2 (87.7, 107.1) |
| **Connection** |  |  |  |  |  |
| Mean (SD) | 91.8 (11.6) | 90.2 (10.5) | 91.6 (12.4) | 91.7 (11.6) | 93.9 (11.8) |
| Range | 68.1, 125.7 | 68.1, 120.8 | 68.1, 120.8 | 68.1, 120.8 | 68.1, 125.7 |
| Median (IQR) | 90.5 (83.1, 98.5) | 88.6 (83.1, 98.5) | 90.5 (83.1, 98.5) | 90.5 (83.1, 99.6) | 94.4 (86.7, 102.7) |
| **Coping** |  |  |  |  |  |
| Mean (SD) | 93.0 (10.7) | 91.2 (11.0) | 92.0 (10.9) | 93.6 (9.7) | 95.2 (10.8) |
| Range | 69.0, 127.4 | 72.2, 127.4 | 69.0, 122.5 | 75.6, 117.8 | 69.0, 117.8 |
| Median (IQR) | 91.0 (87.0, 99.5) | 91.0 (83.1, 99.5) | 91.0 (87.0, 99.5) | 91.0 (87.0, 99.5) | 95.2 (87.0, 103.9) |
| **Fulfilment** |  |  |  |  |  |
| Mean (SD) | 92.0 (10.5) | 91.6 (10.0) | 91.5 (10.5) | 90.8 (10.7) | 94.3 (10.4) |
| Range | 68.3, 120.5 | 72.9, 115.3 | 68.3, 117.9 | 68.3, 120.5 | 70.6, 117.9 |
| Median (IQR) | 92.2 (84.8, 99.8) | 89.7 (84.8, 97.2) | 92.2 (84.8, 97.2) | 89.7 (84.2, 97.2) | 94.7 (87.3, 99.8) |
| **Happiness** |  |  |  |  |  |
| Mean (SD) | 95.6 (10.9) | 95.3 (10.5) | 95.2 (10.6) | 94.6 (10.7) | 97.3 (11.8) |
| Range | 68.8, 121.9 | 72.6, 119.4 | 72.6, 119.4 | 68.8, 117.0 | 68.8, 121.9 |
| Median (IQR) | 96.0 (87.2, 102.9) | 94.9 (87.2, 102.9) | 93.8 (87.2, 100.6) | 96.0 (87.2, 102.9) | 98.3 (89.3, 105.2) |
| **Health** |  |  |  |  |  |
| Mean (SD) | 93.7 (11.6) | 93.8 (12.4) | 93.1 (11.9) | 92.9 (10.5) | 94.9 (11.6) |
| Range | 69.2, 126.7 | 69.2, 126.7 | 73.1, 122.9 | 73.1, 119.1 | 69.2, 126.7 |
| Median (IQR) | 92.5 (84.8, 100.2) | 96.4 (84.8, 100.2) | 92.5 (84.8, 100.2) | 92.5 (84.8, 100.2) | 94.4 (88.7, 104.0) |
| **Sleep** |  |  |  |  |  |
| Mean (SD) | 94.5 (12.2) | 94.6 (12.4) | 94.8 (11.8) | 93.7 (11.7) | 95.0 (13.0) |
| Range | 73.2, 124.3 | 73.2, 124.3 | 73.2, 117.9 | 73.2, 117.9 | 73.2, 124.3 |
| Median (IQR) | 94.9 (84.4, 101.6) | 91.4 (84.4, 104.1) | 94.9 (84.4, 105.0) | 94.9 (84.4, 101.6) | 94.9 (84.4, 105.0) |
| AYM: Activate Your Mood, MYM: Mind Your Mood, FH: Finding Happiness, SD: Standard Deviation, IQR: Inter-quartile Range, PHQ-9: Patient Health Questionnaire-9, GAD-7: Generalized Anxiety Disorder-7, SWEMWBS: Short Warwick-Edinburgh Mental Wellbeing Scale, WPAI: Work Productivity and Activity Impairment. | | | | | |
