# Supplementary material for: Feasibility and Preliminary Efficacy of Digital Interventions for Depressive Symptoms in Working Adults: Multiarm Randomized Controlled Trial
Source: JMIR Form Res. 2023 Jun 16;7:e41590. doi: 10.2196/41590 (PMC10337296; doi:10.2196/41590)
Supplement: Multimedia Appendix 3 [file formative_v7i1e41590_app3.docx]

| **Multimedia Appendix 3.** Reported reasons for session and activity non completion, obtained at t1 | | | | |  |
| --- | --- | --- | --- | --- | --- |
|  | Overall | Study arm | | |  |
|  |  | AYM | FH | MYM |  |
| **Reasons for session non completion, n (%)** |  |  |  |  |  |
| I wasn't able to create an Unmind account, or the instructions were unclear | 4 (4.3) | 0 (0) | 0 (0) | 4 (16.0) |  |
| I didn't have time to finish the Series | 23 (25.0) | 13 (30.2) | 5 (20.8) | 5 (20.0) |  |
| I forgot that I was taking part in the study | 7 (7.6) | 6 (14.0) | 1 (4.2) | 0 (0) |  |
| I lost motivation to take part in the study | 14 (15.2) | 5 (11.6) | 6 (25.0) | 3 (12.0) |  |
| I didn't enjoy the Unmind Series or I found it boring | 8 (8.7) | 3 (7.0) | 2 (8.3) | 3 (12.0) |  |
| I didn't feel like it was helpful in any way | 8 (8.7) | 3 (7.0) | 3 (12.5) | 2 (8.0) |  |
| I experienced technical difficulties | 13 (14.1) | 3 (7.0) | 5 (20.8) | 5 (20.0) |  |
| I didn't have access to the internet | 4 (4.3) | 1 (2.3) | 0 (0) | 3 (12.0) |  |
| Other | 11 (12.0) | 9 (20.9) | 2 (8.3) | 0 (0) |  |
| **Reasons for activity non completion, n (%)** |  |  |  |  |  |
| I wasn't able to create an Unmind account, or the instructions were unclear | 7 (3.8) | 1 (1.6) | 2 (3.3) | 4 (6.6) |  |
| I didn't have time to do the tasks | 38 (20.7) | 12 (19.0) | 9 (15.0) | 17 (27.9) |  |
| I forgot to do the tasks | 35 (19.0) | 12 (19.0) | 9 (15.0) | 14 (23.0) |  |
| I lost motivation to do the tasks | 31 (16.8) | 11 (17.5) | 12 (20.0) | 8 (13.1) |  |
| I didn't enjoy the tasks or I found them boring | 15 (8.2) | 4 (6.3) | 6 (10.0) | 5 (8.2) |  |
| I didn't feel like the tasks were helpful in any way | 18 (9.8) | 7 (11.1) | 9 (15.0) | 2 (3.3) |  |
| I experienced technical difficulties | 11 (6.0) | 3 (4.8) | 4 (6.7) | 4 (6.6) |  |
| I didn't have access to the internet | 6 (3.3) | 1 (1.6) | 2 (3.3) | 3 (4.9) |  |
| I didn't receive the resources via email | 8 (4.3) | 3 (4.8) | 3 (5.0) | 2 (3.3) |  |
| Other | 15 (8.2) | 9 (14.3) | 4 (6.7) | 2 (3.3) |  |
| AYM: Activate Your Mood, MYM: Mind Your Mood, FH: Finding Happiness | | | | | |
