# Supplementary material for: Feasibility and Preliminary Efficacy of Digital Interventions for Depressive Symptoms in Working Adults: Multiarm Randomized Controlled Trial
Source: JMIR Form Res. 2023 Jun 16;7:e41590. doi: 10.2196/41590 (PMC10337296; doi:10.2196/41590)
Supplement: Multimedia Appendix 4 [file formative_v7i1e41590_app4.docx]

**Multimedia Appendix 4.**


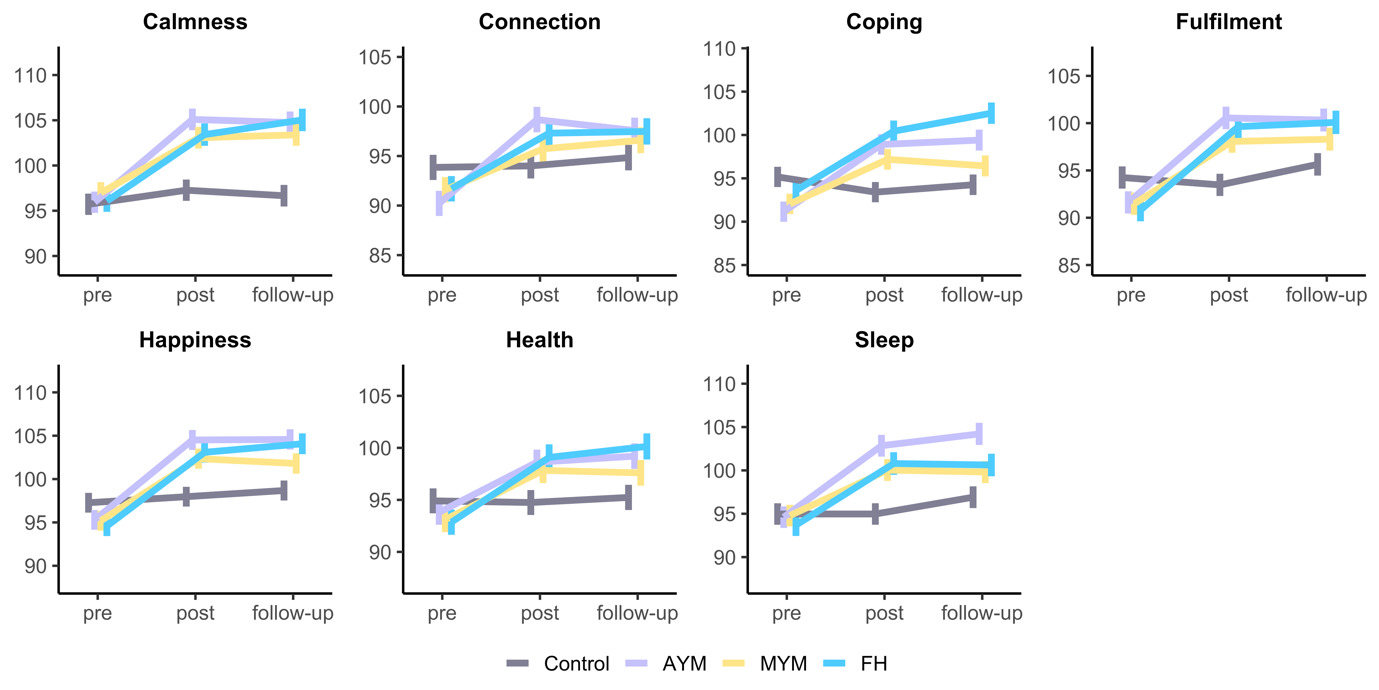


Plots of estimated marginal means obtained from LME models for the seven subscales of the Unmind Index
