# Supplementary material for: Feasibility and Preliminary Efficacy of Digital Interventions for Depressive Symptoms in Working Adults: Multiarm Randomized Controlled Trial
Source: JMIR Form Res. 2023 Jun 16;7:e41590. doi: 10.2196/41590 (PMC10337296; doi:10.2196/41590)
Supplement: Multimedia Appendix 5 [file formative_v7i1e41590_app5.docx]

| **Multimedia Appendix 5.** Contrasts and between-group (intervention versus control) effect size calculations from linear mixed-effects models (LMMs) applied to each of the seven Unmind Index subscales | | | | | | |
| --- | --- | --- | --- | --- | --- | --- |
| Outcome T1 minus t0 | | | | T2 minus t0 | | |
|  | Estimate (SE, 95%CI) | P value | Hedges g (95% CI) | Estimate (SE, 95%CI) | P value | Hedges g (95% CI) |
| **Calmness** | |  |  |  |  |  |
| AYM | 7.60 (1.76; 4.34, 10.86) | <0.001** | 0.636 (0.354, 0.919) | 7.87 (1.70; 4.55, 11.18) | <0.001** | 0.648, (0.365, 0.931) |
| MYM | 4.53 (1.68; 1.26, 7.80) | 0.007** | 0.379 (0.101, 0.658) | 5.47 (1.70; 2.15, 8.78) | <0.001** | 0.451, (0.171, 0.731) |
| FH | 5.80 (1.71; 2.47, 9.12) | <0.001** | 0.478 (0.197, 0.759) | 8.03 (1.72; 4.68, 11.38) | <0.001** | 0.658, (0.373, 0.943) |
| **Connection** | |  |  |  |  |  |
| AYM | 8.31 (1.58; 5.22, 11.39) | <0.001** | 0.736 (0.451, 1.021) | 6.37 (1.61; 3.23, 9.50) | <0.001** | 0.555, (0.274, 0.836) |
| MYM | 3.97 (1.59; 0.88, 7.06) | 0.012* | 0.352 (0.073, 0.63) | 4.01 (1.61; 0.87, 7.15) | 0.013* | 0.35, (0.071, 0.628) |
| FH | 8.03 (1.72; 4.68, 11.38) | <0.001** | 0.658, (0.373, 0.943) | 5.46 (1.61; 2.31, 8.61) | <0.001** | 0.476 (0.195, 0.757) |
| **Coping** | |  |  |  |  |  |
| AYM | 9.51 (1.65; 6.29, 12.72) | <0.001** | 0.808 (0.521, 1.095) | 9.16 (1.67; 5.90, 12.43) | <0.001** | 0.767, (0.481, 1.053) |
| MYM | 6.90 (1.65; 3.68, 10.12) | <0.001** | 0.587 (0.304, 0.869) | 5.29 (1.67; 2.03, 8.56) | 0.002** | 0.444, (0.164, 0.724) |
| FH | 8.59 (1.68; 5.31, 11.87) | <0.001** | 0.72 (0.434, 1.006) | 9.82 (1.69; 6.52, 13.12) | <0.001** | 0.817, (0.529, 1.106) |
| **Fulfilment** | |  |  |  |  |  |
| AYM | 9.71 (1.59; 6.61, 12.81) | <0.001** | 0.856 (0.568, 1.144) | 7.32 (1.61; 4.17, 10.47) | <0.001** | 0.635, (0.353, 0.918) |
| MYM | 7.36 (1.59; 4.25, 10.47) | <0.001** | 0.649 (0.365, 0.932) | 5.43 (1.62; 2.28, 8.59) | <0.001** | 0.472, (0.192, 0.753) |
| FH | 9.60 (1.62; 6.44, 12.77) | <0.001** | 0.834 (0.545, 1.123) | 7.89 (1.63; 4.70, 11.07) | <0.001** | 0.68, (0.395, 0.965) |
| **Happiness** | |  |  |  |  |  |
| AYM | 8.51 (1.59; 5.42, 11.61) | <0.001** | 0.751 (0.466, 1.037) | 7.87 (1.61; 4.72, 11.01) | <0.001** | 0.683, (0.4, 0.967) |
| MYM | 6.46 (1.59; 3.36, 9.57) | <0.001** | 0.57 (0.288, 0.853) | 5.19 (1.61; 2.04, 8.34) | 0.001** | 0.452, (0.172, 0.732) |
| FH | 7.81 (1.62; 4.65, 10.98) | <0.001** | 0.68 (0.394, 0.965) | 8.09 (1.63; 4.90, 11.27) | <0.001** | 0.698, (0.413, 0.984) |
| **Health** | |  |  |  |  |  |
| AYM | 4.97 (1.46; 2.12, 7.82) | <0.001** | 0.477 (0.197, 0.756) | 5.07 (1.48; 2.17, 7.97) | <0.001** | 0.478, (0.198, 0.758) |
| MYM | 4.89 (1.46; 2.03, 7.75) | <0.001** | 0.469 (0.188, 0.749) | 4.16 (1.49; 1.26, 7.06) | 0.005** | 0.393, (0.114, 0.672) |
| FH | 6.39 (1.49; 3.48, 9.30) | <0.001** | 0.603 (0.32, 0.886) | 6.95 (1.50; 4.02, 9.89) | <0.001** | 0.652, (0.367, 0.936) |
| **Sleep** | |  |  |  |  |  |
| AYM | 8.24 (1.69; 4.95, 11.54) | <0.001** | 0.683 (0.399, 0.967) | 7.67 (1.72; 4.32, 11.02) | <0.001** | 0.626, (0.343, 0.908) |
| MYM | 5.25 (1.69; 1.95, 8.56) | 0.002** | 0.435 (0.156, 0.715) | 3.10 (1.72; -0.27, 6.44) | 0.073 | 0.252, (-0.025, 0.53) |
| FH | 7.10 (1.72; 3.74, 10.47) | <0.001** | 0.58 (0.297, 0.863) | 5.00 (1.74; 1.609, 8.38) | 0.004** | 0.405, (0.125, 0.685) |
| AYM: Activate Your Mood, MYM: Mind Your Mood, FH: Finding Happiness **p<0.01 (highly significant) *p<0.05 | | | | | | |
